# A phase-stable dual-comb interferometer

**DOI:** 10.1038/s41467-018-05509-6

**Published:** 2018-08-02

**Authors:** Zaijun Chen, Ming Yan, Theodor W. Hänsch, Nathalie Picqué

**Affiliations:** 1 Max-Planck-Institut für Quantenoptik, Hans-Kopfermann-Straße 1, 85748 Garching, Germany; 2 Ludwig-Maximilians-Universität München, Fakultät für Physik, Schellingstr. 4/III, 80799 München, Germany

## Abstract

Laser frequency combs emit a spectrum with hundreds of thousands of evenly spaced phase-coherent narrow lines. A comb-enabled instrument, the dual-comb interferometer, exploits interference between two frequency combs and attracts considerable interest in precision spectroscopy and sensing, distance metrology, tomography, telecommunications, etc. Mutual coherence between the two combs over the measurement time is a pre-requisite to interferometry, although it is instrumentally challenging. At best, the mutual coherence reaches about 1 s. Computer-based phase-correction techniques, which often lead to artifacts and worsened precision, must be implemented for longer averaging times. Here with feed-forward relative stabilization of the carrier-envelope offset frequencies, we experimentally realize a mutual coherence over times approaching 2000 s, more than three orders of magnitude longer than that of state-of-the-art dual-comb systems. An illustration is given with near-infrared Fourier transform molecular spectroscopy with two combs of slightly different repetition frequencies. Our technique without phase correction can be implemented with any frequency comb generator including microresonators or semiconductor lasers.

## Abstract (web summary)

Dual-comb interferometry promises to be a high-precision metrology technique, but is hindered by short coherence times that limit the maximum number of averages. Here, Chen et al. achieve mutual coherence times of almost 2000 s by feed-forward stabilization of the carrier-envelope offsets.

## Introduction

The performance of laser frequency combs^1^ has been constantly perfected to meet scientific challenges such as optical-clock comparisons^2^ or low-noise microwave generation^3^. For about a decade, novel applications involving time-domain interference between the two frequency combs have emerged and hold promise for enhancing the precision of interferometric measurements, as encountered in spectroscopy and sensing^4–7^, distance metrology^8^, tomography^9^, telecommunications^10^, etc. With a dual-comb system, a type of two-beam interferometer, the phase difference is in most of the implementations automatically and periodically scanned by means of two asynchronous trains of pulses. Although such systems have a potential for precisions directly set by atomic clocks, they still fail in many aspects to compete with mechanical interferometers. Unfortunately, it is still challenging to control the relative timing and phase fluctuations between the two combs and therefore to keep them coherent over extended measurement times. Conversely, the precise control of the phase difference in a two-beam interferometer involving a moveable mirror has indeed been perfected over decades and the mutual coherence between the two arms of the interferometer can be maintained over tens of hours^11^ in standard laboratory environments.

The most powerful approach for establishing mutual coherence between two frequency combs has been to lock, with fast intra-cavity actuators, each comb to the same pair of cavity-stabilized continuous-wave lasers with hertz-level linewidth. In this way, mutual coherence times of the order of 1 s, determined by the linewidth of the continuous-wave lasers, have been achieved and linear-phase correction enhances the effective averaging times to tens of minutes^7^. Alternatively, schemes correcting the relative fluctuations with analog electronics^6^, digital processing^12^, or computer algorithms^13^ permit measurements, even with free-running lasers. Another current trend is to design systems with built-in passive mutual coherence^5,14^. None of these solutions reaches the overall performance of the cavity-locked systems. Although already technically involved, mutual coherence times of about 1 s represent a strong limitation: numerical phase correction is required to reach the averaging times of several tens or hundreds of minutes. Phase-correction techniques have been widely documented in the context of Michelson-based Fourier transform spectroscopy^15^ and are straightforwardly transposable to dual-comb spectroscopy. Unfortunately, such techniques are complex and may generate computational errors and artifacts in the spectra^16,17^. Moreover, implementing them is not always feasible: emission spectra composed of scarce lines, as encountered in coherent Raman^18^ or two-photon excitation^19^ dual-comb spectroscopy, are known to be particularly challenging to phase correct^20^. In Michelson-based Fourier transform spectroscopy, a proper interferometer design, such as that of the Connes-type interferometers^21,22^, makes phase correction superfluous. Connes-type interferometers have been widely recognized by the molecular-spectroscopy community as instruments of superior performance for high-resolution Doppler-limited spectroscopy.

With the increasing number of foreseen applications for highly precise dual-comb systems, for instance to spectroscopic measurements of very weak lines, to Doppler-free broadband spectroscopy^19^, to precise measurements of refractive indices^23^, or to distance monitoring^24^ between formations of spacecrafts, breaking the barrier of 1 s for the interferometer coherence times and enabling dual-comb spectroscopy without phase correction is crucial. Excellent performance has already been reported with all types of spectrometers for direct frequency comb spectroscopy, including Michelson-based Fourier transform spectrometers^25–27^ and dispersive spectrometers^28^. Dual-comb spectroscopy has the distinguishing advantage, though, that the resolution only derives from the measurement time, rather than from geometry (e.g., the path difference excursion in a Michelson interferometer or the grating length in a dispersive spectrograph). Therefore—in principle—the resolution in a single non-interleaved dual-comb spectrum is fundamentally limited by the comb line spacing only, rather than by the instrumental resolution of a spectrometer. This implies that the coherence time of the dual-comb interferometer is sufficiently long to resolve the individual comb lines. Extended mutual coherence times will for instance enable the measurement of broadband spectra with combs of narrow line spacing (<1 MHz) in a single continuous measurement. Such an accomplishment will accelerate the development of Doppler-free multiplex spectroscopy^19^ and will open up exciting prospects for precision spectroscopy and metrology over broad spectral spans.

In this article, we introduce a new concept for maintaining the coherence in a dual-comb system. Using feed-forward control of the relative carrier-envelope offset frequency of the lasers, we experimentally demonstrate a mutual coherence time of 1860 s, more than three orders of magnitude longer than the current state of the art. Furthermore, we do not observe any indications that we have reached a limit, suggesting that phase control in a dual-comb interferometer can be of arbitrarily long duration.

## Results

### Principle of feed-forward dual-comb interferometry

**Fig. 1:**
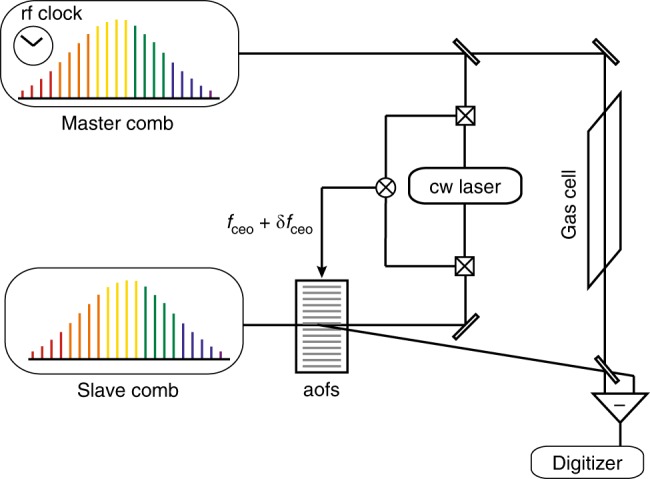
Sketch of the principle of feed-forward dual-comb spectroscopy. A self-referenced master comb provides long-term stability and the slave frequency comb follows the drifts of the master comb, providing high-bandwidth mutual coherence. The difference in carrier-envelope offset frequencies is kept constant by a feed-forward stabilization scheme that acts, through an acousto-optic frequency shifter (aofs), on the carrier-envelope offset frequency *f*_ceo_ + *δf*_ceo_ of the slave beam. The beam of the master comb interogates the sample and beats with the beam emerging from the acousto-optic frequency shifter in the first-order of diffraction beam. The optical signal is detected with a balanced differential detector and is digitized

We propose and implement a technique of dual-comb interferometry, which demonstrates mutual coherence times of 1860 s, without any indications that a limit is reached. We use feed-forward adjustment of the relative carrier-envelope offset frequencies of the two combs with an external actuator, which permits very fast response time without locking electronics and may be used on any types of frequency comb generators. Feed-forward control of a laser is a technique known for fast response time and low noise, as successfully demonstrated for the frequency stabilization of a continuous-wave laser^29^, for the stabilization of a mode-locked laser to a Fabry-Perot cavity^30^ or for carrier-envelope offset lock of a frequency comb^31^. In our scheme (Fig. 1), one frequency comb (master), of repetition frequency *f*_rep_ and carrier-envelope offset *f*_ceo_, is stabilized against a radio-frequency clock using the traditional self-referenced technique, which provides frequency accuracy over long timescales. This stabilization does not contribute to the establishment of the mutual coherence between the two combs, which would work as well if the master comb was free-running. However, for a serious assessment of any instrumental artifacts and systematic effects induced by our feed-forward technique, a high precision is required. The second comb generator (slave), of slightly different repetition frequency *f*_rep_ + *δf*_rep_, with *δf*_rep_ small compared to *f*_rep_, and of carrier-envelope offset *f*_ceo_ + *δf*_ceo_, follows the rapidly varying instabilities of the first master comb. Two beat notes, each between one line of the master comb and one line of the slave comb, serve as indicators of the relative fluctuations between the combs and are maintained at fixed frequency offsets. One beat note generates the error signal for feeding an acousto-optic frequency shifter at the output of the slave comb: all the spectral lines in the first-order diffracted beam of the slave comb are shifted in frequency by the same amount. Relative carrier-envelope offset control is therefore achieved with a bandwidth of several hundreds of kilohertz. The second beat note locks the repetition frequency of the slave comb through a slow feedback loop, which translates one of the cavity mirror of the slave comb by means of a piezo-electric transducer. More details may be found in Methods. The choice of the actuators and of the way to generate the error signals may be adapted to the type of employed frequency comb generators: for instance, combining an electro-optic phase modulator and an acousto-optic frequency shifter may lead to even faster response times.

### Near-infrared feed-forward dual-comb spectroscopy

We illustrate the performance of our interferometer with a setup dedicated to near-infrared dual-comb spectroscopy (Supplementary Fig. 1). Two commercial femtosecond erbium-doped amplified fiber lasers emitting around 190 THz are used. Their repetition frequencies are such that *f*_rep_ = 100 MHz and *δf*_rep_ = 100 Hz. The outputs of the combs are spectrally broadened in highly nonlinear fibers to span the region from 166 to 245 THz. The master comb interrogates a single-pass cell filled with a gas at low pressure, while the first-order diffracted beam of the slave comb serves as local oscillator. The two combs are combined on a beam-mixer. For improved signal-to-noise ratio in a selected spectral region, the optical signal may be spectrally filtered with home-made grating filters of tunable central wavelength and spectral width. A differential detector detects the two outputs of the interferometer. The time-domain interference signal is digitized with a data acquisition board.

**Fig. 2:**
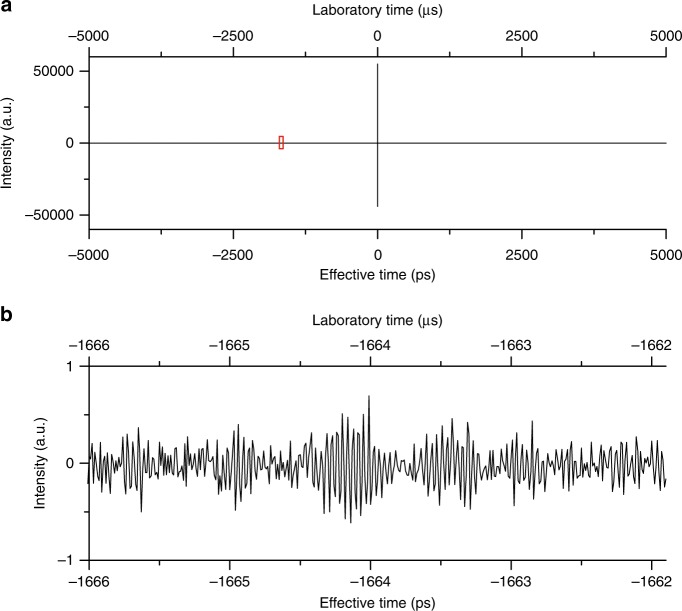
Time-domain interferogram. In the laboratory time frame, the interferograms repeat with a period of 1/*δf*_rep_ = 10 ms, which corresponds to optical delays of 1/*f*_rep_ = 10 ns. **a** In all, 186 000 consecutive interferograms are averaged in the time domain over a span of 10 ms, resulting in a total laboratory time of 1860 s. **b** Magnified view of the region surrounded by a red rectangle in **a** on a *y*-scale expanded 55 000-fold. The signal is due to the characteristic interferometric modulation induced by the molecular lines

An interferogram of acetylene in the region of emission of the oscillators (182–202 THz) is shown in Fig. 2 over the entire range of optical delays of 1/*f*_rep_ = 10 ns. The interferogram results from 186 000 averages over a total measurement time of 1860 s. We can choose to average the individual interferograms in the time domain or the spectra in the frequency domain, the first approach has the advantage of a significantly reduced data file size with easier storage and shorter computation time. When we continuously average the interferograms in the time domain, we do not perform any operations on the raw data other than summing them. We then compute the complex spectrum by Fourier transformation and derive the phase and amplitude of the resulting spectrum (Supplementary Fig. 2). For any averaging times, the signal-to-noise ratio in such time-domain-averaged spectrum is systematically 2% smaller than when we average the individual amplitude spectra.

**Fig. 3:**
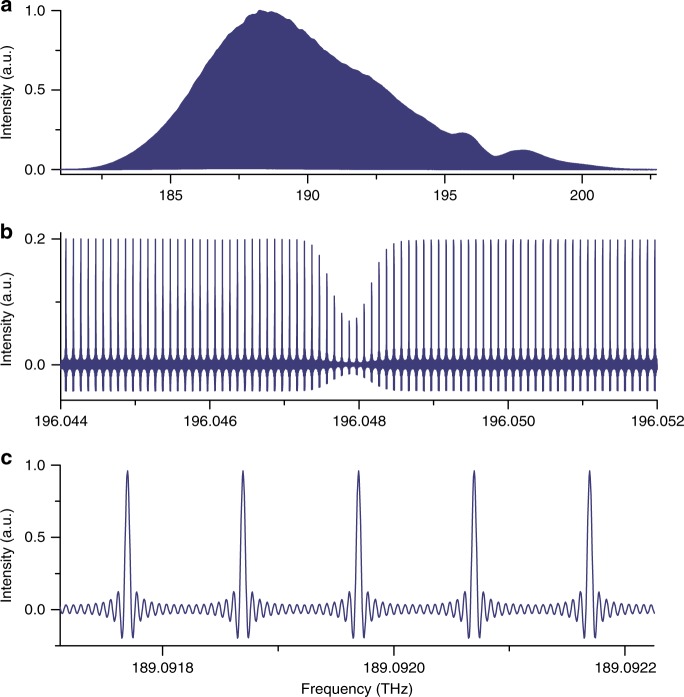
Spectrum around 190 THz with resolved comb lines, measured within 1860 s. **a** Entire apodized spectrum across the entire domain of emission of the laser oscillators: more than 200 000 individual comb lines spanning 20 THz are resolved. **b** Magnified unapodized representation of a. showing the *P*(7) Doppler-broadened line of the *ν*_1_ + *ν*_3_ band of ^12^C_2_H_2_ sampled by the comb lines of 100 MHz line spacing. The Doppler full width at half maximum of the *P*(7) line at 295 K is near 473 MHz. **c** Magnified unapodized representation of **a** showing five individual comb lines with with their cardinal sine instrumental line shape

A spectrum with resolved comb lines, resulting from a measurement time of 1860 s, is shown in Fig. 3. Six thousand interferograms, of 0.31 s duration each, have been averaged. In the spectrum, more than 200 000 individual comb lines are resolved across 20 THz. The observed full width at half maximum of the comb lines is set by the measurement time of an individual interferogram to 3.5 Hz in the radio-frequency domain. Each comb line (Fig. 3c) appears as a cardinal sine, the expected instrumental line shape in a non-apodized spectrum. In our previous report^6^, this instrumental line shape was washed out by residual phase fluctuations. For a measurement time of 1860 s (Supplementary Fig. 2), the signal-to-noise ratio in the spectrum culminates at 8550 around 188.2 THz and the average signal-to-noise ratio across the entire span of 20 THz is 3850. The resulting figure of merit, calculated for the average signal-to-noise ratio, is therefore 1.8 × 10^7^ Hz^1/2^. Our value of the figure of merit is slightly higher but of the same order of magnitude than that reported in ref. ^7^. As in any experiments of dual-comb spectroscopy, a limitation to the sensitivity is the need to restrict the power falling onto the detectors to avoid artifacts induced by the nonlinearities. In our experiment, the total average power on one photo-detector is limited to 50 µW, an amount that is experimentally determined as the threshold for measurable systematic effects. Detector nonlinearities in Fourier transform spectroscopy^32,33^, and their manifestations^34^ in dual-comb spectroscopy, are widely documented in the scientific literature. Small residual nonlinearities generate subtle line shifts, on the megahertz scale, and line profile distortions, which for instance affect the line intensities on the percent scale.

**Fig. 4:**
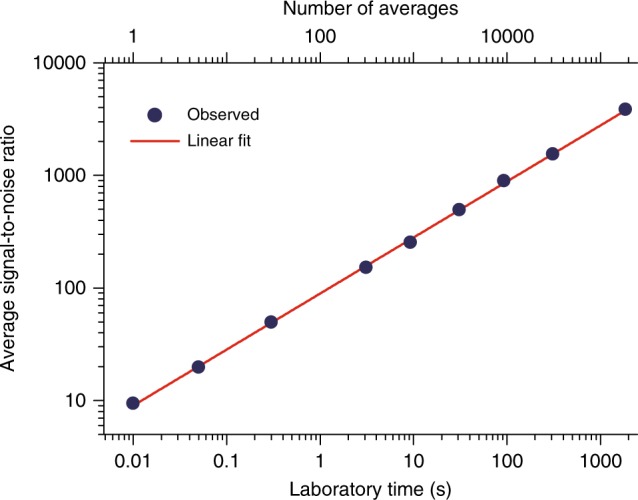
Evolution of the signal-to-noise ratio in the spectra with the measurement time. The interferograms are averaged in the time domain and Fourier transformed with a resolution of 100 MHz. The average signal-to-noise ratio in the spectra is plotted for different times of averaging. A linear fit with a slope of 0.503(5) indicates that the signal-to-noise is proportional to the square root of the measurement time, which is expected for coherent averaging. The number in parentheses is the standard deviation in units of the last quoted digit. The trend has not reached its limit at the maximum measurement time of 1860 s

Figure 4 displays the evolution of the average signal-to-noise ratio with time (or number of averages). It increases with the square root of the measurement time, showing that the mutual coherence between the two combs is preserved throughout. The same behavior is observed near 180 and 230 THz. The maximum averaging time that we report, 1860 s, is a technical limitation due to our data acquisition system. No saturation in the trend of the increasing signal-to-noise ratio is observed though. This suggests that with a dedicated data acquisition system, coherence over longer averaging times will be achieved.

**Fig. 5:**
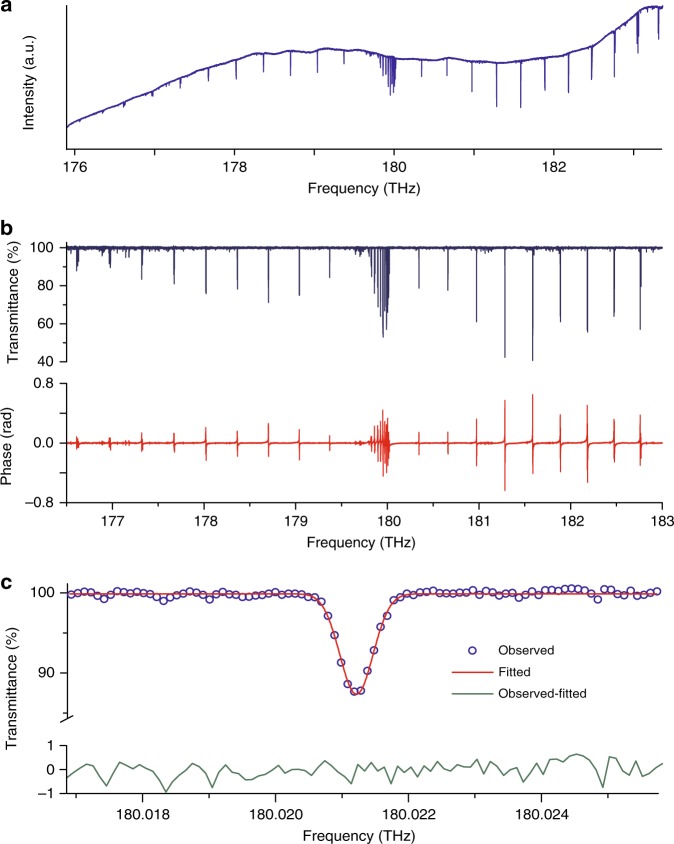
Experimental dual-comb spectrum at a resolution of 100 MHz in the 180-THz region. **a** The amplitude spectrum of the 2*ν*_3_ band of ^12^CH_4_ spans 9 THz and it is measured within 14.46 s. **b** Transmittance (blue) and dispersion (red) spectra. **c** Portion of the transmittance spectrum (blue open dots, labeled observed), magnifying the *Q*(1) line with about 13% of absorption at line center. The Doppler full width at half maximum of the *Q*(1) line at 295 K is approximately 554 MHz. The experimental profile is satisfactorily fitted by a Doppler line shape (red line, labeled fitted). The standard deviation of the residuals observed-fitted is 0.3%, at the noise level

Good signal-to-noise ratios are achieved all across the region spectrally broadened by nonlinear fibers. As an example, the amplitude and phase spectra of methane in the region of the 2*ν*_3_ band around 180 THz are shown (Fig. 5a, b) at 100-MHz resolution. The filtered spectral span is 175–184 THz and the recording time is 14.46 s. The signal-to-noise ratio is at best 770 around 183.2 THz and the average signal-to-noise ratio is 465, which leads to a figure of merit of 1.1 × 10^7^ Hz^1/2^. Another representation with resolved comb lines is displayed in Supplementary Fig. 3.

We determine the line positions in our self-calibrated spectra by fitting Doppler profiles of fixed width to the experimental transitions. Because of the long averaging time, of the narrow width of the optical comb lines and of the high mutual coherence of the dual-comb system, the instrumental line shape that convolves the profiles, can be neglected. The residuals of the fit do not show any systematic signatures, as exemplified in Supplementary Fig. 2c with the *P*(17), *P*(16), and *P*(15) lines of the *ν*_1_ + *ν*_*3*_ band of ^12^C_2_H_2_ and in Fig. 5c with the *Q*(1)*F*_2_ line of the 2*ν*_3_ band of ^12^CH_4_. For the *ν*_1_ + *ν*_3_ band of ^12^C_2_H_2_, we compare the positions of 11 of our experimental lines, for which the self-induced pressure shift has been measured in^35^, to accurate sub-Doppler saturated absorption measurements^36,37^. The mean value of the discrepancies between our measurements and those of refs. ^36^,^37^, respectively, is 110 and 44 kHz, respectively, with a standard deviation of 275 and 285 kHz, respectively. Our accuracy is primarily determined by the statistical uncertainty. Additional details may be found in Supplementary Table 1 (and Supplementary Table 2 for two lines in ^12^CH_4_).

## Discussion

With our demonstration of a dual-comb interferometer with long mutual coherence times, new opportunities for broadband metrology are opened up. Moreover, our technique is expected to significantly improve the performance of dual-comb interferometers in spectral regions, like the mid-infrared domain or the ultraviolet region, where their development still faces numerous challenges. The hertz-line-width continuous-wave lasers that would allow for mutual coherence times of the order of 1 s are challenging to develop in these regions^38^. Fast intra-cavity actuators for the two degrees of freedom of the combs are not straightforwardly available with any type of frequency comb generator either. Dual-comb systems involving light sources based on nonlinear frequency conversion, such as synchronously pumped optical parametric oscillators, may take advantage of the simplifications brought by our technique. Furthermore, microresonators^39–42^ and semiconductor lasers^43,44^ have recently demonstrated an exciting prospect for compact dual-comb spectrometers. Feed-forward control may provide the appropriate tool for the fast control of the mutual coherence of such interferometers.

## Methods

### Detailed experimental setup

Two erbium-doped fiber laser oscillators, each with three output ports, are used to generate two frequency combs, which we call master comb and slave comb. The principle is sketched in Fig. 1, and Supplementary Fig. 1 gives additional details on the technical implementation. Their repetition frequency is around 100 MHz and it may be adjusted and controlled by translating an intra-cavity mirror mounted on a piezo-electric transducer, changing the laser cavity length. The pulse duration is about 90 fs and the center frequency 190 THz. Each oscillator has an output emitting an average power of about 10 mW. The two additional output ports feed two erbium-doped fiber amplifiers, each providing up to 300 mW. One of the amplifiers of the master comb is used for the traditional self-referencing scheme: the spectrum is broadened in a nonlinear fiber and the carrier-envelope offset frequency is detected using a *f*-2*f* interferometer. Both the carrier-envelope offset frequency and the repetition frequency of the master comb are locked to the 10-MHz radio-frequency signal of an active hydrogen maser delivering a fractional instability of 2 × 10^−13^ at 1 s. All the electronic instruments (synthesizers, counters, digitizers, etc) in our experiment are synchronized to this 10-MHz clock signal. In our experiments, we lock the repetition frequency of the master comb at precisely *f*_rep_ = 100 MHz. The repetition frequency of the slave comb *f*_rep_ + *δf*_rep_ is chosen such that *δf*_rep_ = 100 Hz, leading to an interferometric free spectral range of 50 THz. For interferometry, the output of the second amplifier of the master comb and that of one of the amplifers of the slave comb are spectrally broadened in nonlinear fibers of normal dispersion to a span covering from 166 to 245 THz.

The coherence between the master and the slave combs is maintained by forcing the slave comb to follow in real time the fast residual timing and phase fluctuations of the self-referenced master comb. As in many other setups of dual-comb spectroscopy with fiber lasers^6,7,12^, two radio-frequency beat notes, originating from two different pairs of individual lines of the two combs are used as gauges for the relative fluctuations between the two combs. The beat notes are produced with optical signals after the spectral broadening in the nonlinear fibers in order to account for as many noise sources as possible. A beam-splitter extracts a small percentage of the power of the master comb beam, while most of the power remains available for the interferometer described below. After spectral broadening, an acousto-optic frequency shifter diffracts the beam of the slave comb. About 70% of the power is transferred to the first-order diffracted beam, which is used for interferometry with stable phase scans and for the slow feedback loop on the repetition frequency (both described later), while the remaining non-diffracted zero-order beam is used to synthesize the signal that monitors and corrects by feed-forward control the fast relative fluctuations between the master and the slave combs.

### Feed-forward control

A beat note between one line of the master comb and one line of the slave combs is produced. We use an erbium-doped continuous-wave laser at 189 THz, with a reasonable passive stability, as an intermediate oscillator. The continuous-wave laser beats with each comb. An individual line of each comb is therefore isolated and the beat signal is interferometrically amplified by the larger power of the continuous-wave laser. By mixing the two beat notes, of a line of the master comb with the continuous-wave laser and of a line of the slave comb with the same continuous-wave laser, the contribution of the continuous-wave laser cancels out and an electric signal at the frequency difference between one line of the master comb and one line of the slave comb is produced. This electrical signal monitors the relative fluctuations of the two erbium-doped fiber combs at a given optical frequency. It is mixed with the radio-frequency signal generator, amplified and it directly drives an acousto-optic frequency shifter. In the first-order diffracted beam of the acousto-optic frequency shifter, the frequency of all the comb lines is shifted by the same amount. This quantity contains a constant arbitrary radio-frequency shift and a smaller acoustic shift that follows in real time the relative fluctuations of the slave and master comb. This is equivalent to an adjustment of the carrier-envelope offset frequency of the slave comb relative to that of the master comb. As no locking electronics is required, the bandwidth of the corrections is only limited by that of the acousto-optic frequency shifter. Here the response time of our frequency shifter and related electronics is 550 ns (which leads to a bandwidth of about 300 kHz, considering that the bandwidth is one-sixth of the inverse of the response time^45^) and its rise time is 70 ns. On the timescale of our measurements (30 min), the fibered acousto-optic frequency shifter provides the required compensations without adding intensity noise. For significantly longer measurement times or for other laser systems, a free-space double-passed acousto-optic frequency shifter, an electro-optic phase modulator^46^, or the combination of the fast feed-forward control and a slow feedback loop that would prevent drifts of *δf*_ceo_ may represent better alternatives of implementation. We choose δ*f*_ceo_ as an integer multiple of δ*f*_rep_; the time-domain interferometric waveforms are then precisely periodic, without burst-to-burst phase-shift, and they can be efficiently averaged in the time domain.

### Stabilization of the relative repetition frequencies

For the relative stabilization of the second degree of freedom of the slave comb, a radio-frequency beat note between one line of the master comb and one line of the slave comb is produced at 195 THz using the same technique as that described above. It is compared to the signal of a signal generator. The resulting error signal is fed-back to the piezo-electric transducer controlling the cavity length of the slave laser with a low bandwidth (<1 kHz). Depending on the type of laser frequency combs that are used (available actuators and noise sources), other implementations of the entire concept of relative stabilization may be devised.

### Interferometer

Our dual-comb interferometer is dedicated to multiheterodyne spectroscopy. The beam of the master comb interacts with a gas sample in a single-pass cell. It is combined on a beam-mixer with the beam of the slave comb diffracted in the first order by the acousto-optic modulator. The interferometric signals at the outputs of the beam-mixer have the same amplitude and opposite phases. They are both detected and subtracted by a differential detector. The electric signal is then filtered, amplified, digitized and, if needed, averaged. The digitization is performed synchronously to the master comb repetition frequency for efficient time-domain averaging. A complex Fourier transform is computed. The phase and the amplitude of the spectrum are retrieved and are displayed in our figures (Fig. 5b, Supplementary Fig. 2b). The radio-frequency scale is converted to an optical scale by using the values of the repetition frequency and carrier-envelope offset frequency of the master laser, which are counted during the measurement.

### Experimental conditions for the spectra with gas samples

The single-pass cell which is used in all experiments has a length of 70 cm. The temperature of the laboratory is 295 K. The gas pressure is measured with capacitance manometers. In the spectra shown in Fig. 3 and Supplementary Fig. 2, the pressure of acetylene, in natural abundance, is 195.2 Pa. In the spectra shown in Fig. 5 and Supplementary Fig. 3, the pressure of methane, in natural abundance, is 1067 Pa.

### Code availability

The simple Matlab program used to compute the Fourier transforms is available from the corresponding author upon reasonable request.

### Data availability

The data that support the plots within this paper and other findings of this study are available from the corresponding author upon reasonable request.

## Electronic supplementary material

Supplementary Information
