# Supplementary material for: A phase-stable dual-comb interferometer
Source: Nat Commun. 2018 Aug 2;9:3035. doi: 10.1038/s41467-018-05509-6 (PMC6072769; doi:10.1038/s41467-018-05509-6)
Supplement: Supplementary file 1 — Supplementary Information [file 41467_2018_5509_MOESM1_ESM.pdf]

Supplementary Information to "A phase-stable dual-comb interferometer"

Zaijun Chen[1,2], Ming Yan[1,2], Theodor W. Hänsch[1,2], Nathalie Picqué[1,2]
1. Max-Planck-Institut für Quantenoptik, Hans-Kopfermann-Straße 1, 85748 Garching, Germany
2. Ludwig-Maximilians-Universität München, Fakultät für Physik, Schellingstr. 4/III, 80799 München, Germany

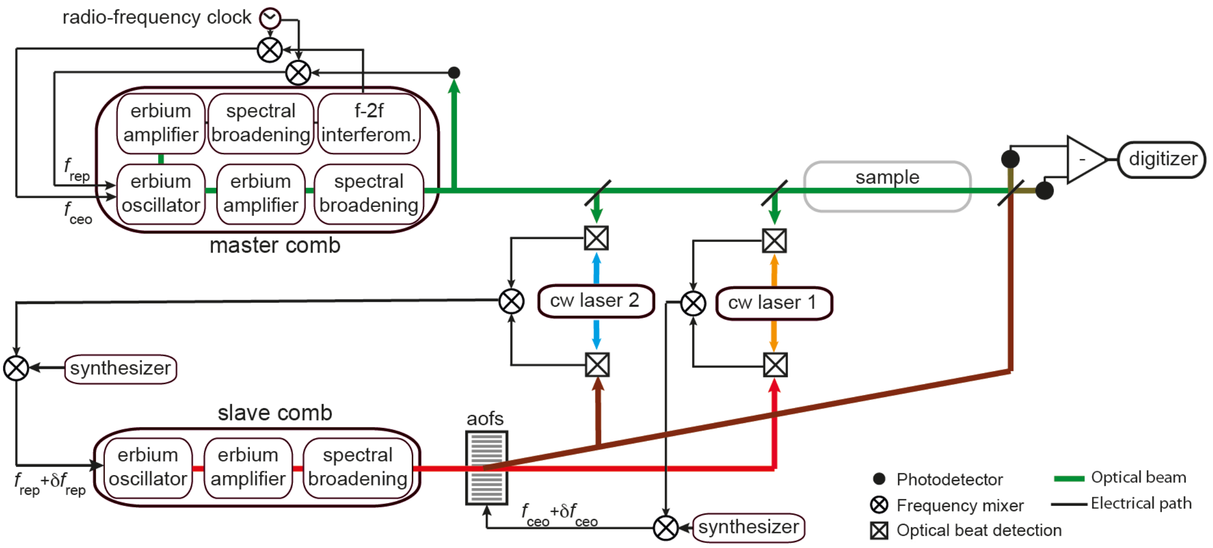

Supplementary Figure 1. **Experimental set-up of near-infrared feed-forward dual-comb spectroscopy.**

Two amplified erbium-doped fiber lasers emit at a central frequency of 190 THz with a pulse repetition frequency of about f_{rep}=100 MHz. One comb, called master comb, is self-referenced against a radio-frequency clock. Its repetition frequency and carrier-envelope offset frequency are f_{rep} and f_{ceo}, respectively. The mutual coherence in the dual-comb interferometer is maintained over extended time periods (>30 min) by feed-forward control of the relative carrier-envelope-offset frequency δf_{ceo}, using an acousto-optic frequency shifter (aofs) on the beam of the second comb (called slave comb). A slow feedback loop adjusts the relative repetition frequency δf_{rep} by modifying the length of the slave laser oscillator. The error signals for the feed-forward and feed-back corrections are provided by the beat signals between two pairs of comb lines, one from each comb. Two continuous-wave lasers (cw laser 1 and cw laser 2) serve as intermediate oscillators to generate the two required beat signals. For dual-comb spectroscopy, the master comb interrogates the sample and its beam is combined with that of the slave comb diffracted by the acousto-optic frequency shifter. The time-domain interferogram, which is the difference of the two beat signals after the beam mixer, is digitized. Details are given in the Methods section.

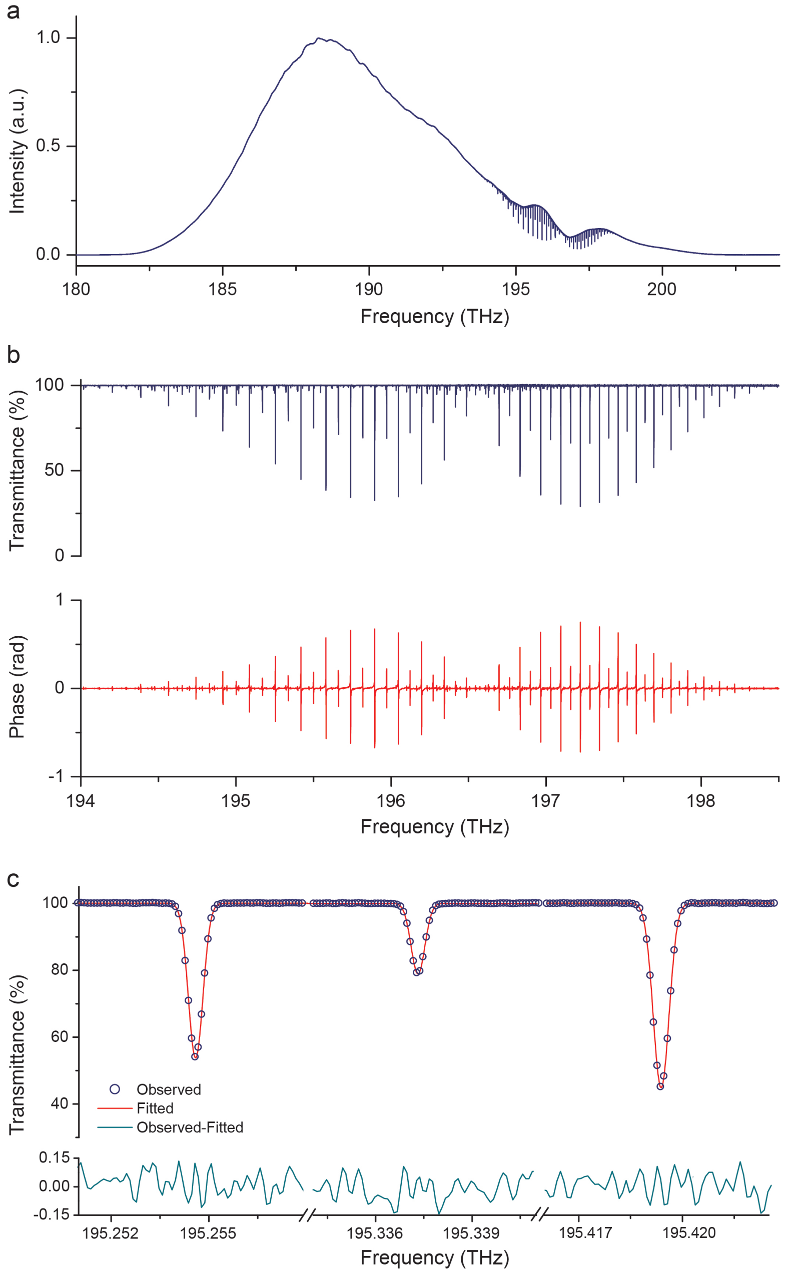

Supplementary Figure 2. Experimental spectrum in the region of the v_1+v_3 combination band of $^{12}C_2H_2$ with a resolution of 100 MHz corresponding exactly to the comb line spacing.

a. The entire spectrum spans 20 THz and it is measured within 1860 s. **b.** Transmittance and dispersion spectra of the v_1+v_3 band of $^{12}C_2H_2$. The baseline has been corrected using a polynomial fit **c**. Magnified representation of the transmittance spectrum in b. showing the the $P(17)$, $P(16)$ and $P(15)$ line of the v_1+v_3 band of $^{12}C_2H_2$. The transmittance y-scale only goes down to 30%. A Doppler profile (red line) fits the experimental spectrum (blue dots) data is fitted with a Gaussian profile. The standard deviation of the residuals (green, on an amplified y-scale) between the experimental data and the fit is 0.06%.

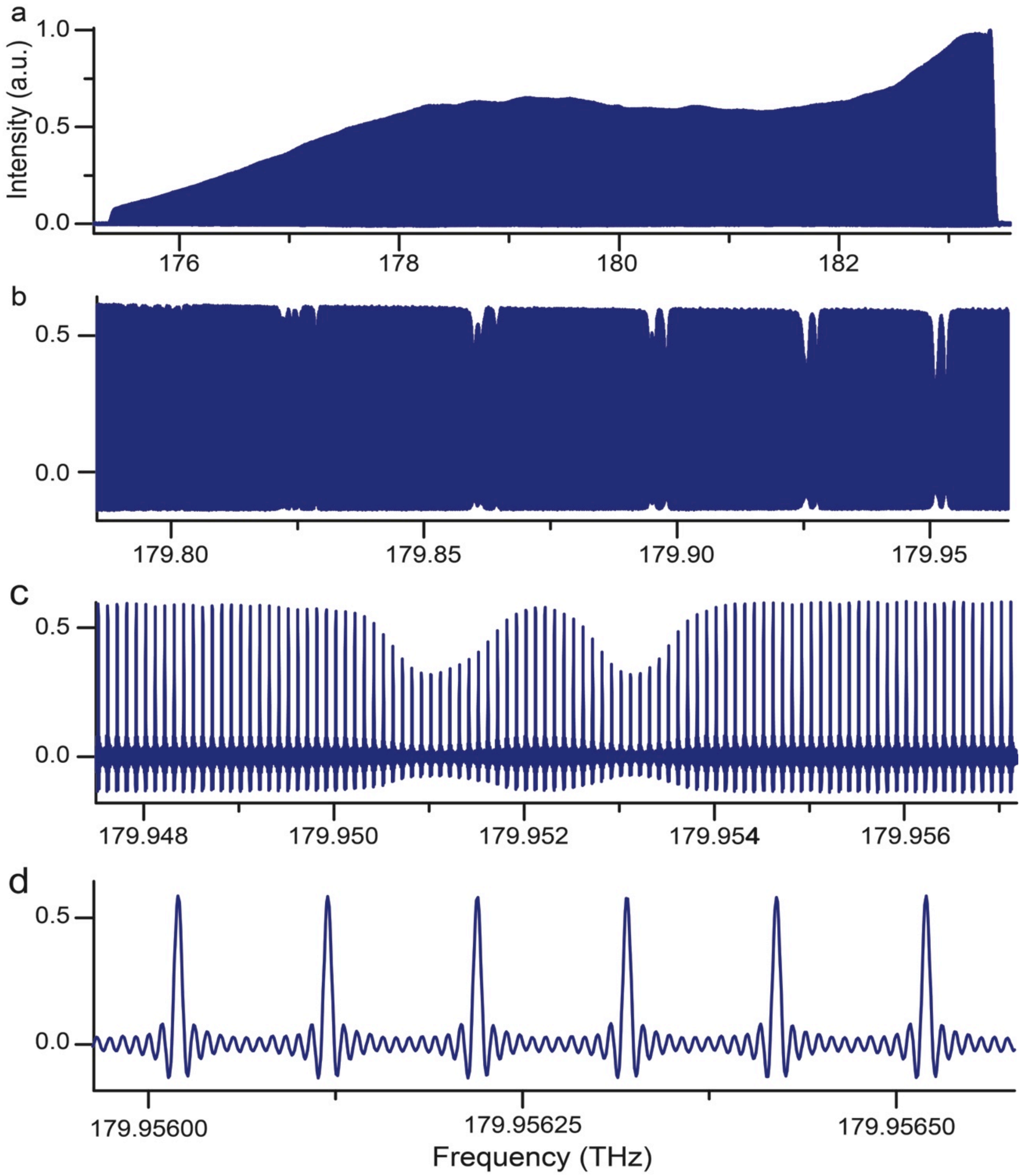

Supplementary Figure 3. Experimental dual-comb spectrum with resolved comb lines around 180 THz. The $2\nu_3$ band of $^{12}CH_4$ is sampled within a multiplex measurement time of 14.46 s. The spectrum is displayed apodized in a. and unapodized in b.,c.,d. **a**. The entire span covers 9 THz. **b**. Portion of the spectrum shown in a. with some of the multiplets in the *Q* branch of the $2\nu_3$ band of $^{12}CH_4$. **c.** Magnified view of the *Q*(6) manifold. **d**. Magnified view of six individual comb lines.

Assignment	Frequency (MHz)						
	This work	Pressure shift at 195.2 Pa [1]	This work extrapolated to zero pressure	[2]	[3]	This work-[2]	This work-[3]
P(14)	195 500 510.1(5)	-0.40(4)	195 500 510.5(5)	195 500 510.62(15)	195 500 510.7477(101)	-0.1	-0.2
P(13)	195 580 979.3(3)	-0.40(4)	195 580 979.7(3)	195 580 979.28(13)	195 580 979.3711(102)	0.4	0.3
P(10)	195 817 848.2(4)	-0.42(4)	195 817 848.6(4)	195 817 848.23(15)	195 817 848.3823(105)	0.4	0.2
P(6)	196 123 037.7(4)	-0.40(7)	196 123 038.1(4)	196 123 038.51(15)	196 123 038.5214(103)	-0.4	-0.4
P(5)	196 197 428.0(2)	-0.42(4)	196 197 428.4(2)	196 197 428.20(13)	196 197 428.3457(102)	0.2	0.1
P(4)	196 271 052.2(5)	-0.42(4)	196 271 052.6(5)	196 271 052.46(14)	196 271 052.5819(103)	0.1	0.0
P(3)	196 343 910.0(4)	-0.40(4)	196 343 910.4(4)	196 343 910.06(14)	196 343 910.0006(127)	0.3	0.4
R(1)	196 696 653.0(4)	-0.20(4)	196 696 653.2(4)	-	196 696 652.9203(100)	-	0.3
R(7)	197 094 394.3(3)	-0.42(4)	197 094 394.7(3)	197 094 394.83(15)	197 094 395.0333(104)	-0.1	-0.3
R(11)	197 343 962.2(4)	-0.57(4)	197 343 962.8(4)	197 343 962.37(15)	-	0.4	-
R(17)	197 694 759.3(3)	-0.81(4)	197 694 760.1(3)	197 694 760.15(15)	-	-0.1	-

Supplementary Table 1. Center frequencies of the Doppler-broadened lines in the $\nu_1+\nu_3$ band of $^{12}C_2H_2$ measured in this work (retrieved from the spectrum shown in Supplementary Fig. 2 measured within 31 minutes) along with a comparison with the absolute frequency measurements reported in [2] and [3] by Doppler-free saturated absorption spectroscopy. Our experimental line positions at zero pressure are corrected (fourth column) for the pressure shift using the values measured by [1]. We only provide frequencies for the lines for which the pressure shift had been determined and accurate measurements for comparisons are available. The number within parentheses is the uncertainty, including statistical and systematic effects, in units of the last digit.

Assignment	Frequency (MHz)				
	This work	Pressure shift at 1067 Pa [4]	This work extrapolated to zero pressure	[5]	This work - [5]
R(0)	180 345 060.9 (25)	-5.51(3)	180 345 066.4(25)	180 345 065.08(37)	1.3
Q(1)	180 021 248.7 (32)	-6.11(6)	180 021 254.8(32)	180 021 253.10(61)	1.7

Supplementary Table 2. Center frequencies of two Doppler-broadened lines in the $2\nu_3$ band of $^{12}CH_4$ measured in this work (retrieved from the spectrum shown in Figure 5, measured within 14.46 s). The fourth column gives the frequencies extrapolated to zero pressure, corrected using the pressure shifts measured in [4]. These two lines are the only ones for which accurate measurements (based on Doppler-free saturated absorption spectroscopy) are available in the literature [5]. The number within parentheses is the uncertainty, including statistical and systematic effects, in units of the last digit.

Supplementary References.

[1] Swann, W. C. & Gilbert, S. L. Pressure-induced shift and broadening of 1510-1540 nm acetylene wavelength calibration lines, *J. Opt. Soc. Am. B* **17**, 1263-1270 (2000).
[2] Nakagawa, K., de Labachelerie, M., Awaji, Y. & Kourogi, M. Accurate optical frequency atlas of the 1.5-μm bands of acetylene, *J. Opt. Soc. Am. B* **13**. 2708-2714 (1996).
[3] Edwards, C.S., Barwood, G.P., Margolis, H.S., Gill, P. & Rowley, W.R.C. High-precision frequency measurements of the $\nu_1+\nu_3$ combination band of $^{12}C_2H_2$ in the 1.5 μm region, *J. Mol. Spectrosc.* **234**, 143-148 (2005).
[4] Lyulin, O.M., Perevalov, V.I., Morino, I., Yokota, T. & Watanabe T., Measurements of self-broadening and self-pressure-induced shift parameters of the methane spectral lines in the 5556–6166 cm^{-1} range, *J. Quant. Spectrosc. Radiat. Transf.* **112,** 531-539 (2011).
[5] Ishibashi, C., Kourogi, M., Imai, K., Widiyatmoko, B. & Sasada H. Absolute frequency measurement of the saturated absorption lines of methane in the 1.66 μm region, *Opt. Commun.* **161**, 223-226 (1999).
